# A BCL2 promoter polymorphism rs2279115 is not associated with BCL2 protein expression or patient survival in breast cancer patients

**DOI:** 10.1186/2193-1801-1-38

**Published:** 2012-10-23

**Authors:** Claire J Searle, Ian W Brock, Simon S Cross, Sabapathy P Balasubramanian, Malcolm WR Reed, Angela Cox

**Affiliations:** 1 Department of Oncology, CR-UK/YCR Sheffield Cancer Research Centre, University of Sheffield Medical School, Beech Hill Road, Sheffield, UK; 2 Department of Clinical Genetics, Chapel Allerton Hospital, Chapeltown Road, Leeds, UK; 3 Academic Unit of Pathology, Department of Neuroscience, University of Sheffield Medical School, Beech Hill Road, Sheffield, UK; 4 Academic Unit of Surgical Oncology, CR-UK/YCR Sheffield Cancer Research Centre, University of Sheffield Medical School, Beech Hill Road, Sheffield, UK

**Keywords:** Breast cancer, BCL2, rs2279115, Survival, SNP

## Abstract

The B-cell CLL/lymphoma 2 (BCL2) gene family encodes pro- and anti-apoptotic proteins that are critical regulators of programmed cell death. Higher levels of BCL2 expression in breast tumours have been shown to be an independent prognostic factor for improved survival from breast cancer. The promoter single nucleotide polymorphism (SNP) rs2279115 has been associated with both BCL2 expression and patient survival. The aim of this study was to attempt to replicate these observations in a cohort of 1015 UK women with breast cancer, and to compare genotype frequencies in cases and controls. In this study, 1015 breast cancer cases and 1034 control subjects were genotyped for the rs2279115 SNP by 5’ nuclease PCR. Paraffin embedded tumour tissue for 342 case subjects was assembled into tissue microarrays, and the level of expression of BCL2 was established by immunohistochemistry. Kaplan Meier survival curves and Cox Proportional Hazards models were used to examine the effect of genotype on patient survival. The effect of SNP genotype on tumour BCL2 protein levels and breast cancer susceptibility was assessed by logistic regression. In this study higher BCL2 expression was significantly associated with improved survival from breast cancer (p = 0.015), in keeping with previous reports. The SNP rs2279115 was not found to be associated with tumour expression of BCL2, (p = 0.77), and neither was it associated with case/control status (p = 0.25). There was no significant association between the SNP and overall survival (p = 0.75). In conclusion, we found that higher tumour BCL2 expression is associated with improved survival from breast cancer, in keeping with previous studies. However, in contrast to a previous report, the promoter SNP rs2279115 was not associated with BCL2 expression or overall survival from breast cancer.

## Background

The balance between cell proliferation and levels of apoptosis is frequently disrupted in tumours, with tumorigenesis being promoted by both the loss of pro-apoptotic signals and the gain of anti-apoptotic mechanisms (Hanahan & Weinberg 2000; Hanahan & Weinberg 2011). The BCL2 family of proteins plays a crucial role in these processes, by integrating the complex pathways incorporating pro- and anti-apoptotic signals at the mitochondrial membrane (Tsujimoto 2002). The BCL-2 family can be categorised into anti-apoptotic and two pro-apoptotic subgroups. The anti-apoptotic members include BCL2 and Bcl-xL. The pro-apoptotic members can be divided into a “multi-BH domain” group including Bax and Bak and a BH3-only subgroup (Adams & Cory 2002). However, BCL2 itself seems to act as both an oncogene and a tumour suppressor gene in different tumour types. For example, higher levels of tumour BCL2 expression are associated with poor patient survival from chronic lymphocytic leukaemia (CLL), but with improved survival from breast and colon cancer (Faderl et al. 2002) (Buglioni et al. 1999) (Callagy et al. 2008).

The *BCL2* gene consists of three exons and two promoters; it is located on chromosome 18q21.3. The SNP (rs2279115) is located in the inhibitory P2 promotor of the *BCL2* gene (Park et al. 2004). The C allele in comparison to the A allele displayed significantly increased inhibition of *BCL2* promoter activity and binding of nuclear proteins (Nuckel et al. 2007). In keeping with these findings BCL2 protein expression in B cells from CLL patients carrying the AA genotype was significantly increased compared with CC genotypes (Nuckel et al. 2007). This relationship was also demonstrated in relation to lymph node negative breast cancer in one previous study (Bachmann et al. 2007). In this study higher expression of BCL2 was associated with the A-allele (p = 0.044) and Kaplan-Meier survival analysis revealed a significant association of the AA genotype with improved survival (p = 0.030). This relationship has also been demonstrated in oropharyngeal squamous cell carcinoma (Lehnerdt et al. 2009) where rs2279115 was significantly associated with BCL2 expression (p = 0.008) and with overall survival (p = 0.0247). This trend was also demonstrated in renal cancer (Hirata et al. 2009).

Many studies have clearly demonstrated that increased BCL2 expression is associated with improved outcome from breast cancer (Yang et al. 2003) (Callagy et al. 2006) (Callagy et al. 2008)^.^ (Dawson et al. 2010) (Ali et al. 2012). A multivariate analysis incorporating five published studies from 11,212 breast cancer cases strongly supported the independent prognostic significance of BCL2 positivity with improved survival (Hazard Ratio (HR) 0.76, 95% Confidence Interval (CI) 0.54-0.74), p <0.001)(Dawson et al. 2010). In addition, expression of BCL2 has been proven to be an independent indicator of favourable prognosis for all types of early-stage breast cancer (Callagy et al. 2008; Dawson et al. 2010).

The aim of this study was to use a cohort of breast cancer cases, from the Sheffield Breast Cancer Study (SBCS) to determine whether there is a relationship between the promoter SNP rs2279115 and tumour protein levels of BCL2, and whether this corresponds to any differences in patient survival. We also confirmed the known association between high levels of tumour BCL2 and improved survival from breast cancer.

## Materials and methods

### Subjects

Between November 1998 and January 2005, 1274 women with breast cancer and 1271 control subjects were enrolled in the SBCS. The design and methodology of this case control study have been previously described (Rafii et al. 2002) (Azmy et al. 2004). Briefly, all subjects were residents of South Yorkshire, UK and were of European descent. The breast cancer cases all had histopathologically confirmed breast cancer. The control subjects were women aged between 50 and 65 attending the Sheffield Mammography Screening Service between September 2000 and January 2004, whose mammograms showed no evidence of breast lesions. The study was approved by the South Sheffield Research Ethics Committee (SSREC/98/137), and the DNA samples were collected with informed consent from subjects for their use in genetic studies of cancer. Paraffin-embedded tumour tissue was requested from the relevant NHS Histopathology Archive for 342 of the subjects recruited above. Pathological data (including tumour grade, morphology and lymph node status) were obtained from medical pathology records and validated (SSC). Immunohistochemical data for the oestrogen receptor (ER), progesterone receptor (PR), HER2 and cytokeratins 5/6 were available (Blows et al. 2010). Data on all-cause mortality and survival was obtained through the Trent Cancer Registry. Median follow-up for breast cancer cases in September 2009 was 21.6 years including 220 deaths.

### Determination of BCL2 rs2279115 genotype

Blood DNA samples were available from 1015 breast cancer subjects and 1034 controls. These were genotyped for the SNP rs2279115 using a Taqman 5’ nuclease PCR assay. The Probe sequence was as follows 5’-CTCCCCAGGAGAGAGACAGGGGAGA[G/T]GGGACGATGAAGGAGCCGGGGACGG-3’, with the FAM probe containing T and the VIC probe containing G. The amplification reaction was performed in a final volume of 5 μL, with 1.0 μL of genomic DNA (10 ng), 0.125 μL of TaqMan™ Genotyping Assay, 2.5 μL of Taqman Genotyping Master Mix, and 2.375 μL of water. The thermo- cycling conditions were as follows: 95°C for 10 min followed by 60 cycles of 92°C for 10 s and 60°C for 1 min. Allelic discrimination was carried out using the ABI 7900HT Sequence Detector (Life Technologies) The overall genotype call rate was 96% (980 cases and 981 controls successfully genotyped), and duplicate concordance based on 133 duplicate samples was 99.25%. The observed control genotype frequencies were consistent with Hardy Weinberg equilibrium (p = 0.76).

### BCL2 immunohistochemistry

**Figure 1:**
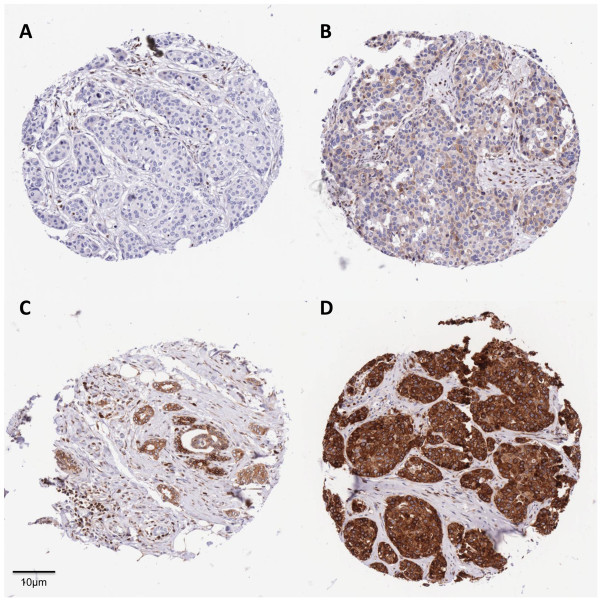
**Immunohistochemical evaluation of BCL2 protein expression in tissue microarrays of breast tumours.** Tissue microarray cores illustrating the BCL2 immunohistochemistry scoring system. Panels **A**-**D** show zero (score 0), low (score 1), moderate (score 2) and high (score 3) BCL2 expression respectively.

Tissue micro arrays were constructed from 342 archived paraffin embedded tumour samples from the cancer cohort. Appropriate regions of tumour (judged by H.&.E staining) were selected from the blocks and 0.6 mm triplicate tissue cores were punched out from these regions using a custom precision instrument (Beecher Instrument Inc., Sun Prairie, US). These were then transferred into recipient paraffin blocks in a specific orientation. 5 μm sections from the array blocks were dried, deparaffinised and rehydrated before blocking endogenous peroxidase with a solution of 2% hydrogen peroxide in methanol. The sections were then subjected to antigen retrieval by microwave treatment in 10 Mm tri-sodium citrate. This was followed by a standard immunohistochemistical staining procedure for BCL2 using a mouse monoclonal anti-human BCL2 antibody (Dako Code M0887) at a dilution of 1:50. The slides containing samples in triplicate were assessed for BCL2 signal intensity (by two independent observers (CJS and IWB) who were blinded to the genetic and clinical data) and scored semi-quantitatively. Each core on the microarray was given a score from zero (no stain) to 3 (high staining) depending on the intensity of the BLC2 staining from the tumour cells (Figure 1). The highest of the three triplicate scores for each tissue sample was used for statistical analysis. Agreement between the two independent observers was good as demonstrated by the Kappa statistic (k = 0.81).

### Statistical analysis

All data was initially entered into a Microsoft Access database and exported to STATA 9.2 for statistical analysis. Data analysis was restricted to the 980 case and 981 control subjects for whom SNP genotypes were successfully called. Breast cancer epidemiological risk factors were assessed by Mann Whitney U test for continuous variables and by Pearson χ^2^ test for binary variables. The relationship between BCL2 protein expression in tumours and SNP genotype, and that between SNP genotype and case/control status was assessed in a logistic regression model with AA as the reference genotype. Kaplan-Meier survival functions and Cox Proportional Hazards Regression models were used to assess the effects of variables on overall survival, using a left-truncated model to adjust for prevalent cases (Azzato et al. 2009). Overall survival was calculated from the date of first diagnosis to confirmed death or last known date of follow-up, up to a maximum of 10 years.

This project was designed and completed in accordance with the REMARK reporting recommendations for tumour MARKer prognostic studies (McShane et al. 2005).

## Results

### Subject characteristics

Table 1 shows the epidemiological characteristics of the cases and controls. There were no significant differences in the percentage of post-menopausal women, age at menarche or age at menopause between the cancer and control groups. However, the women in the control group were slightly younger compared to the cancer group (median interquartile range (IQR) of 57 (53–61) vs 59 (51–68); p =0.0004). Control subjects were younger when first pregnant (median IQR of 23 (20–26) vs 24 (21–27) in the cancer group (p = 1 × 10^-6^), and had more children (median IQR of 2 (2–3) in the control group vs 2 (1–3) in the cancer group; p = 0.01). A higher proportion of cases had a positive family history of breast cancer compared to control subjects (14.5% vs 10.3%; p = 0.005).

**Table 1:** **Breast cancer risk factors in case and control subjects**

Variable		Controls	Cases	***p***values
**Age**	N	981	980	
	Median (IQR)	57 (53–61)	59 (51–68)	p = 0.0004^a^
**Age at menarche**	N	972	962	
	Median (IQR)	13 (12–14)	13 (12–14)	p = 0.83^a^
**Age at menopause**	N	537	517	
	Median (IQR)	50 (47–52)	50(46–52)	p = 0.62^a^
**Age at first pregnancy**	N	878	807	
	Median (IQR)	23 (20–26)	24 (21–27)	p = 0.000001^a^
**Parity**	N	981	980	
	Median (IQR)	2 (2–3)	2(1–3)	p = 0.01^a^
**Menopausal status**	N	981	978	
	post	679 (69.2%)	653 (66.8%)	
	pre	302 (30.8%)	325 (33.2%)	p = 0.25^b^
**Family history of breast cancer**	N	981	980	
	Yes	101 (10.3%)	142 (14.5%)	
	No	880 (89.7%)	838 (85.5%)	p = 0.005^b^

^a^ Mann Whitney U test. ^b^ Pearson χ^*2*^ test. IQR: Inter-quartile range.

Positive family history was defined as the presence of at least one first degree relative with breast cancer.

**Figure 2:**
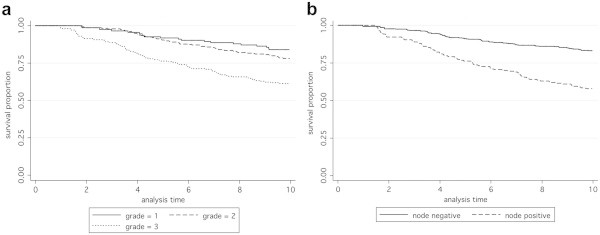
**Kaplan-Meier survival functions according to grade and lymph node status.A**. Overall survival according to tumour grade, based on 769 case subjects with total time at risk 5651.67 years. Numbers at risk at the end of the 10-year analysis period shown were 111 (grade 1), 203 (grade 2), 128 (grade 3). Hazard ratio (95% CI) 1.59 (1.30, 1.96), p = 1.0 × 10^-5^. **B**. Overall survival according to lymph node status, based on 786 case subjects with total time at risk 5808.75 years. Numbers at risk at the end of the 10-year analysis period were 316 (node negative) and 108 (node positive). Hazard ratio (95% CI) 2.59 (1.92, 3.48), p = 3.2 × 10^-10^.

The expected relationships were observed in case subjects for overall survival with tumour grade (p = 1 × 10^-5^) lymph node status (P = 3.2 × 10^-10^) (Figure 2).

### Tumour BCL2 protein levels and overall survival

**Figure 3:**
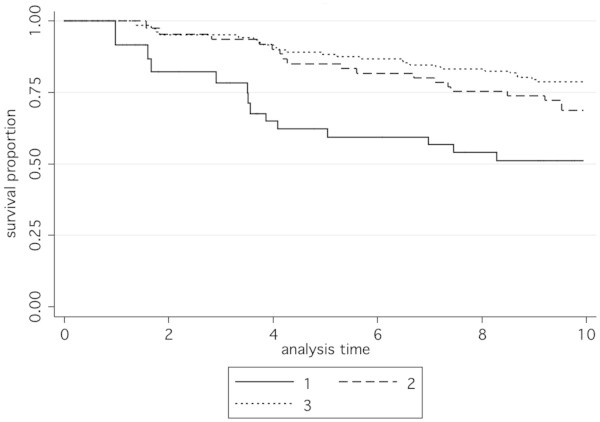
**Kaplan-Meier survival functions according to BCL2 protein expression levels.** Overall survival based on 242 case subjects with total time at risk 1846.10 years. 1: low expression (immunohistochemistry score zero or 1), 2: moderate expression (immunohistochemistry score 2), 3: high expression (immunohistochemistry score 3). Numbers at risk at the end of the 10-year analysis period were 18 (low expression), 39 (moderate expression) and 97 (high expression). Hazard ratio (99.5% CI) 0.69 (0.51, 0.93), p = 0.015.

Following immunohistochemistry to detect BCL2 tumour samples on tissue microarrays, the median intensity score was 3 (range 0–3). Tumours with scores 0–1 were grouped together (low tumour expression of BCL2; n = 35), and the remaining two groups were score 2 (medium tumour expression of BCL2; n = 68) and score 3 (high tumour expression; n = 145). In accordance with previous reports, BCL2 tumour expression was significantly associated with survival (p = 0.015; hazard ratio (95% confidence interval) 0.69 (0.51 to 0.93); Figure 3).

### BCL2 expression level in relation to lymph node status, grade, morphology, ER, PR, HER2, CK5/6 status and rs2279115 genotype

Tumour BCL2 protein expression scores were grouped into binary categories for comparison with standard prognostic variables including tumour grade, nodal status, ER, PR, HER2 and CK5/6 status (Table 2). The binary categories were scores 0–1 being low expression of BCL2 and scores 2–3 being high expression of BCL2. High tumour BCL2 protein expression was associated with lower grade of tumour (p = 4 × 10^-9^), presence of both oestrogen and progesterone receptor in tumours (p = 1 × 10^-14^ and 5 × 10^-5^ respectively), and lack of expression of CK5/6 (p = 0.002). There was no association with nodal involvement (p = 0.54), or morphology (p = 0.68). A higher proportion of HER2 positive tumours were BCL2-low but this was not statistically significant (p = 0.07).

**Table 2:** **Level of BCL2 protein expression according to lymph node status, tumour grade, morphology, ER, PR, HER2 and CK5/6 status**

		Low BCL2 n (%)	High BCL2 n (%)	***p*** value^a^
Node status	No nodal Involvement	20 (64.5)	140 (70.0)	
	Nodal Involvement	11 (35.5)	60 (30.0)	0.54
Grade	1	3 (8.8)	51 (24.9)	
	2	7 (20.6)	114 (55.6)	
	3	24 (70.6)	40 (19.5)	4x10^-9^
Morphology	Ductal	28 (80.0)	159 (75.7)	
	Lobular	2 (5.7)	22 (10.5)	
	Other	5 (14.3)	29 (13.8)	0.68
ER status	Negative	27 (79.4)	31 (15.3)	
	Positive	7 (20.6)	171 (84.7)	1x10^-14^
PR status	Negative	20 (62.5)	53 (26.6)	
	Positive	12 (37.5)	146 (73.4)	5x10^-5^
HER2 status	Negative	29 (82.9)	193 (92.3)	
	Positive	6 (17.1)	16 (7.7)	0.07
CK5/6 status	Negative	23 (67.6)	180 (90.5)	
	Positive	11 (32.4)	19 (9.5)	0.0002

BCL2 immunohistochemistry scores were grouped into low (scores 0–1) and high (scores 2–3) ^a^ Pearson χ^2^ test.

There was no association between the level of expression of BCL2 and rs2279115 genotype (OR (95% CI) 0.83 (0.38, 1.82) and 1.23 (0.37, 4.10) for AC and CC genotypes, respectively, compared to AA genotype; Table 3.

**Table 3:** **Level of BCL2 protein expression of according to rs2279115 genotype**

Genotype	Low BCL2 n (%)	High BCL2 n (%)	Odds Ratio	95% CI		***p*** value
AA	12 (13.3)	78 (86.7)	1.00			
AC	19 (15.6)	103 (84.4)	0.83	0.38	1.82	0.65
CC	4 (11.1)	32 (88.9)	1.23	0.37	4.10	0.74
TOTAL	35 (100)	213 (100.0)				

BCL2 immunohistochemistry scores were grouped into low (scores 0–1) and high (scores 2–3).

### Effect of rs2279115 on breast cancer susceptibility and overall survival

**Figure 4:**
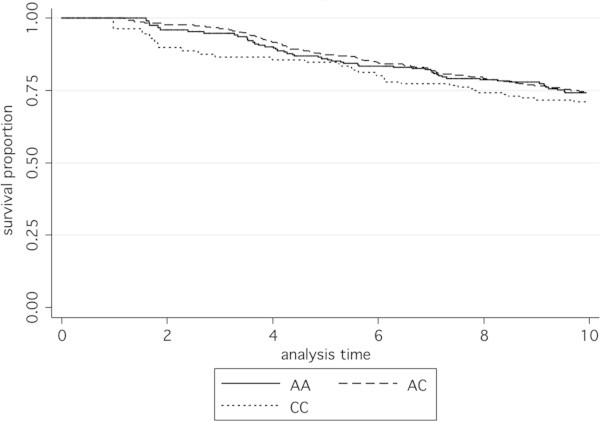
**Kaplan-Meier survival functions according to rs2279115 genotype.** Overall survival based on 934 case subjects with total time at risk 6765.43 years. Numbers at risk at the end of the 10-year analysis period were 158 (AA), 221 (AC), and 102 (CC). Hazard ratio (95% CI) 1.03 (0.86, 1.24), p = 0.75.

**Figure 5:**
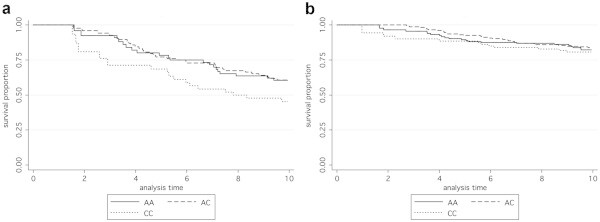
**Kaplan-Meier survival functions in lymph node negative and positive subjects according to rs2279115 genotype.** Overall survival based on 786 subjects with total time at risk 5808.75 years. **A** shows lymph node negative subjects and **B** shows lymph node positive subjects. Numbers at risk at the end of the 10-year analysis period were **A:** 103 (AA), 146 (AC), 79 (CC) and **B:** 38 (AA), 54 (AC), 18 (CC). Hazard ratios (95% CI) for **A** were 0.97 (0.73, 1.30), p = 0.85 and for **B** were 1.20 (0.88, 1.64), p = 0.24.

There was no difference in rs2279115 genotype frequencies between cases and controls; OR (95% CI) were 0.93 (0.76, 1.14) and 0.81 (0.63, 1.04) respectively for AC and CC genotypes compared to AA genotype (Table 4). Furthermore the rs2279115 SNP was not associated with overall survival, HR (95% CI) 1.03 (0.86, 1.24); p = 0.75 (Figure 4). In addition, no statistically significant association was demonstrated between SNP genotype and survival in either lymph node negative or positive subjects (Figure 5; p = 0.85 and 0.24 respectively).

**Table 4:** **Genotype frequencies for SNP rs2279115 in case and control subjects**

Genotype	controls n (%)	cases n (%)	Odds Ratio	95% CI		***p*** value
AA	290 (29.6)	314 (32.0)	1.00			
AC	475 (48.4)	477 (48.7)	0.93	0.76	1.14	0.47
CC	216 (22.0)	189 (19.3)	0.81	0.63	1.04	0.098
TOTAL	981 (100)	980 (100.0)

## Conclusion

The BCL2 family performs a cardinal role in the control of apoptotic pathways, regulating both cell death and cell survival mechanisms by altering mitochondrial membrane permeability and controlling the release of cytochrome c (Reed 1994). Unfavourable outcomes were first reported in relation to inappropriate tumour BCL2 protein expression in follicular lymphoma as a result of a chromosomal translocation t (14,18) (Tsujimoto et al. 1984). It was the break point in this translocation that lead to the discovery of the *BCL2* gene. The data presented here are consistent with previous reports that show that in breast cancer, in contrast to non-Hodgkins lymphoma, higher tumour protein expression of BCL2 is associated with improved survival (Callagy et al. 2006) (Callagy et al. 2008) (Dawson et al. 2010). Anti-apoptotic BCL2 members act as repressors of apoptosis by blocking the release of cytochrome c, whereas pro-apoptotic members act as promoters (Ghobrial et al. 2005). The contrasting effect on survival of tumour BCL2 expression in breast cancer as opposed to non-Hodgkin lymphoma may well be due to the importance of the careful equilibrium between tumour BCL2 protein expression and other pro-apoptotic members such as Bax, rather than on BCL2 tumour protein quantity alone (Reed 1997) (Cory et al. 2003). Unfortunately the exact mechanism that underpins this difference is not fully understood. *In vitro* studies in a variety of different cell types have found that high levels of BCL2 protein expression in tumours can result in striking growth inhibition (Pietenpol et al. 1994). In human breast cancer cell lines there is an inverse correlation between the expression of BCL2 and mutant p53 and that this relationship could lead to down-regulation of BCL2 tumour protein expression (Haldar et al. 1994). Other studies have suggested a function of BCL2 protein in lengthening the cell cycle (O’Reilly et al. 1996) (Knowlton et al. 1998) (Lipponen et al. 1995).

The relationship between tumour BCL2 protein expression and oestrogen has also been widely debated. It has been suggested that the intrinsic and extrinsic pathways which make up the two main routes involved in breast cancer cell apoptosis regulation, are both induced when oestrogen binds to the oestrogen receptor. Both pathways result in the activation of caspase leading finally to apoptosis (Lewis-Wambi & Jordan 2009). Leung and Wang found that a breast cancer cell line treated with the oestrogen 17β-oestradiol resulted in up-regulation of BCL2 mRNA and protein, but down-regulation of Bcl-x(L) mRNA and protein . They did not find this result with other sex hormones. They speculated that different members of the BCL2 family proteins may be regulated through different pathways and that these pathways may be modulated by 17β-oestradiol (Leung & Wang 1999). Tumour BCL2 protein expression status has also been previously strongly associated with PR and ER expression (Nadler et al. 2008) (Lee et al. 1997). Our data are consistent with previous observations that BCL2 is a strong independent prognostic marker for breast cancer survival (Dawson et al. 2010).

The SNP rs2279115 has been associated with BCL2 expression in CLL and breast cancer from node negative patients (Bachmann et al. 2007). The study by Bachmann et al. found that higher expression of BCL2 was associated with the A-allele (P = 0.044) in lymph node negative patients only. This also corresponded to an improved survival in this group (HR (95% CI) 3.2 (1.03,9.93) p = 0.044). Lymph node negative patients who were homozygous for the C allele had a higher risk of death than AA homozygous patients, with heterozygous women being intermediate in risk. In the present data we found no association between rs2279115 and tumour expression of BCL2 in the whole cohort, or when results were subdivided into patient with lymph node positive or lymph node negative disease. We also found no association with survival for the different genotypes. Assuming a baseline survival proportion of 0.84 in lymph node negative cases, our study would have been expected to detect a hazard ratio of 3.2 between homozygous genotypes (as was found by Bachmann et al. 2007), having 80% power to detect hazard ratio of 1.8. However, we are unable to exclude effects smaller than this. It is possible that there may be genotypic effects on survival of similar or smaller magnitude to those of BCL2 expression (Callagy et al. 2008; HR = 1.64); this study is underpowered to detect these.

In conclusion we have no evidence to support the SNP rs2279115 as a prognostic biomarker for breast cancer patients. Higher BCL2 expression has been conclusively proven to correlate with improved survival and further studies are required to explore its use as a prognostic indicator.
